# Supplementary material for: Machine learning the metastable phase diagram of covalently bonded carbon
Source: Nat Commun. 2022 Jun 6;13:3251. doi: 10.1038/s41467-022-30820-8 (PMC9170764; doi:10.1038/s41467-022-30820-8)
Supplement: Supplementary file 1 — Supplementary Information [file 41467_2022_30820_MOESM1_ESM.pdf]

Supplementary Information
Machine Learning the Metastable Phase Diagram of Covalently Bonded Carbon

S. Srinivasan et al.

April 27, 2022

1 Supplementary Methods

1.1 Detailed workflow for construction of phase diagrams

The detailed schematic of our workflow is shown in Supplementary Figure 1. The inputs to our framework are the information about the chemical species, and the temperature and pressure range of interest. Given the chemical information and the range of (T, P) we start our workflow by first identifying the metastable phases using evolutionary structure search.

1.1.1 Evolutionary structure search

Our evolutionary structure search is based on meta-heuristic genetic algorithm, wherein an initial gene pool of crystal structures are randomly guessed and evolved in subsequent generations through genetic mutations or crossover between the fittest structures, which mimics Darwinian evolution.

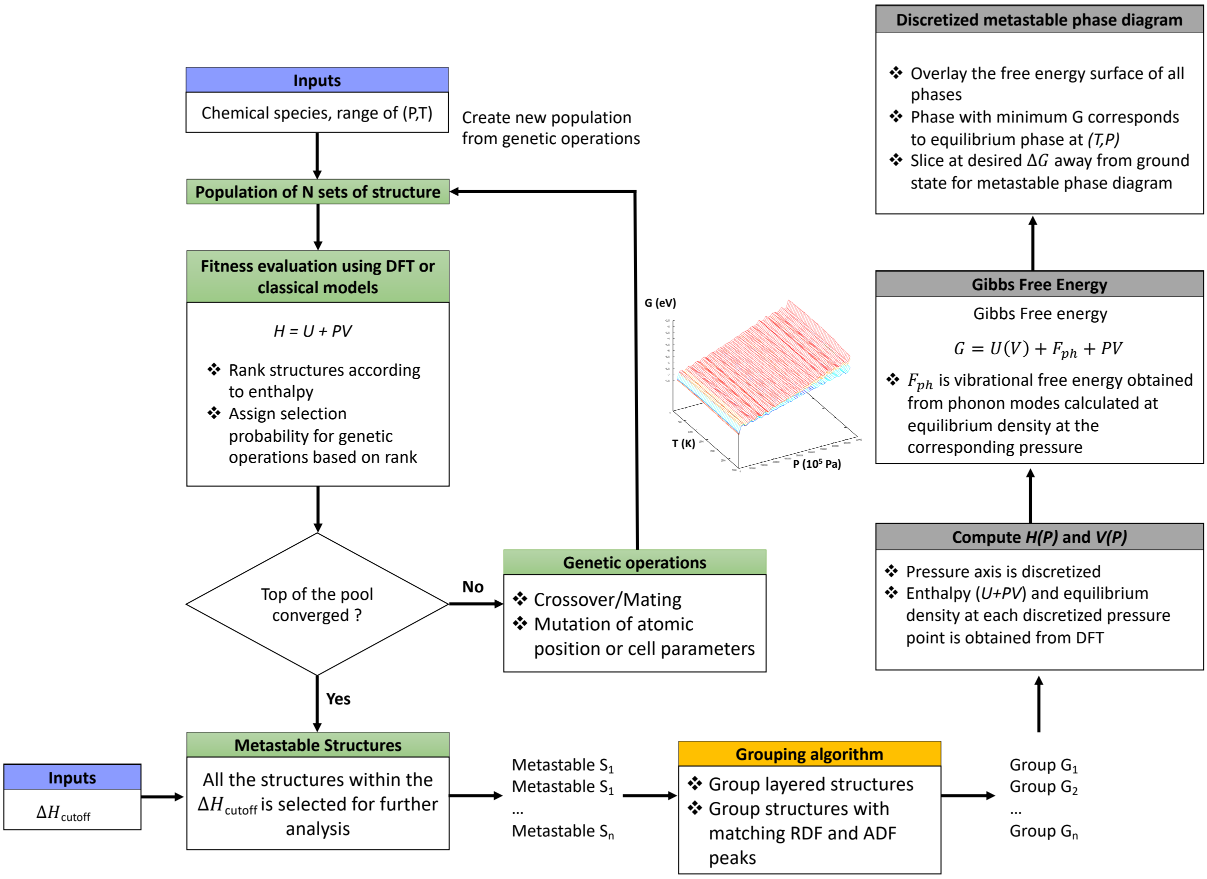

Supplementary Figure 1: Workflow for constructing metastable phase diagrams

We set the size of the gene pool as N=40. We initialize the gene pool with randomly guessed atomic positions and lattice parameters $(a, b, c, \alpha, \beta, \gamma)$, subject to the constrains:

1. no two atoms are closer than 0.5 $\mathring{A}$
2. number of atoms in the unit cells lies between 4 and 20
3. length of the lattice vectors (a, b, c) lie between 2 $\mathring{A}$ and 20$\mathring{A}$
4. lattice angles (α, β, γ) lie between 20°and 160°

Structure search begins by computing the fitness of the initial gene pool structures based on their enthalpies after relaxing under a specified external pressure, with forces computed using density functional theory (DFT) using Perdew, Burke, Ernzerhof approximation with optB86b-vdW [1–3] exchange functional to include the van der Waals interactions.

In addition, we also perform independent evolutionary structure search using the long-range carbon bond-order potential (LCBOP)[4] model. Classical models like LCBOP are cheaper compared to DFT and allows for a quick search over the vast configurational space to identify the far-from-equilibrium metastable structures.

The DFT relaxations are done using the VASP package [5]. LAMMPS package [6] is used to relax structures using LCBOP model. Fitness of each organism (structure) in a given gene pool is evaluated as

$$f_i = \frac{H_i - H_{max}}{H_{min} - H_{max}} \tag{S1}$$

where H_i is the enthalpy of the organism i, H_{max} and H_{min} are the maximum and the minimum enthalpy in the current pool. The gene pool is ranked according to the fitness and parent structures are selected to undergo genetic variations to produce new offspring structures for the subsequent generation of gene pool. The selection probability of each structure is based on the fitness:

$$p_i = \frac{f_i}{\sum_i f_i} \tag{S2}$$

We define three types of genetic operations to build the subsequent generation of structures:

1. **Crossover variation**: This genetic variation involves two parents structures. The offspring structure is generated by slicing the parent structures across a random axis and combing the atoms on one side of the slice with the atoms on the other side in the other parent structure.

2. **Structure mutation**: Structure mutation involves random perturbation of the atomic coordinates and the lattice parameters

3. **Number of atoms mutation**: Atoms are randomly deleted or added in such a way that the constrains on inter-atomic distances and maximum number of atoms allowed.

A new generation offspring structures are generated using the above operations. The probability that a parent structure is subjected to crossover variation, structure mutation and number of atoms mutation are – 0.4, 0.4 and 0.2 respectively. Each of the offspring structure has to pass a redundancy check and satisfy the above mentioned constrains before it can be added to the gene pool of the next generation. Once a new gene pool of 40 structures are obtained, the fitness of the new generation is evaluated and new set of parents are selected based on their probabilities. This cycle is repeated until the difference between the enthalpy of the best and the top $N/8$ structure is less than a tolerance. The tolerance we used for the case of carbon is 20 meV/atom. We build our algorithm based on the modules and function definitions within the *Genetic algorithm for structure and Phase prediction* code [7]. Further details on the algorithm and the genetic variations can be found in Ref. [8, 9].

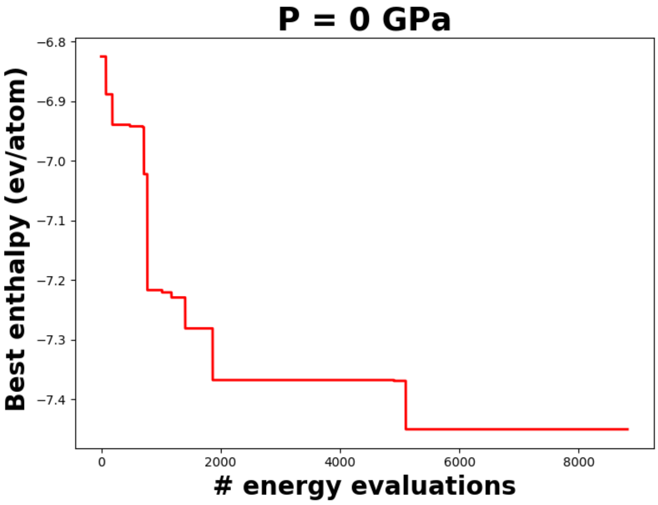

Supplementary Figure 2: Evolution of the best structure in the pool

We perform the independent evolutionary structure searches at $P = 0$ GPa, $P = 10$ GPa & $P = 100$GPa. After convergence, we build a consolidated list of distinct structures ordered according to increasing value

of enthalpies. Only the phases with satisfying $H < H_{ground} + \Delta H_{cut-off}$ are selected for free energy calculations. For carbon, the graphite phase is the experimental ground state with the minimum enthalpy of -7.365 eV/atom (computed using LCBOP model).

1.1.2 Grouping based of RDF and ADF

Some of the candidate structures are structurally very similar and the enthalpies vary only by a small value. For example, in the case carbon, our structure search algorithm predicts hexagonal graphite, orthorhombic graphite and rhombohedral graphite, all which only differ in their stacking patterns and have very similar structural features. Besides at high pressure and temperature conditions, it is highly probable for the layers to slide against each other and change stacking, as can be seen in Figure 3 of the main text. Hence, we group such structures with very high similarity and count them as the same phase. i.e hexagonal graphite, orthorhombic graphite and rhombohedral graphite are considered as "graphite" phase. Only the candidate phase with the least enthalpy within each group is used to compute the free energies. The grouping is done based on the radial distribution function (RDF) and angular distribution function(ADF). Any two structures with matching first two peaks of RDF and ADF are grouped together. We end up with 505 unique groups within 670 meV from the ground state graphite phase. The Crystallographic Information File (CIF) for each grouped structure is provided in a GitHub repository–https://github.com/Srilok/Machine-learning-Metastable-Phase-Diagram

1.2 Phase boundary classification using multiclass support vector machine (MSVM)

Machine learning algorithms like support vector machines (SVMs)[10, 11] which can draw decision boundaries between different classes of inputs are well suited to automate the estimation of phase boundaries given a discrete free energy data set. SVMs are binary classifiers by definition and one has to resort to decomposition techniques like "one-vs-all" or "one-vs-rest"[12] which involves training many classifiers and taking the weighted value of all the output. While such decomposition techniques have been successfully used in the past, it is computationally demanding to train multiple classifiers when there are a large of number phases. Instead, we use a purely multiclass SVM[13–17] (MSVM), using a non-homogeneous 3rd order polynomial kernel, which can classify multiple classes without relying on decomposition techniques. Training only one MSVM classifier reduces the computational time tremendously while maintaining the accuracy of the classifiers. The final equilibrium and metastable phase diagram, generated with the decision boundaries drawn using MSVM, is shown in Figure 2 of the main manuscript.

1.3 Deep Neural Network for screening and baseline model comparison

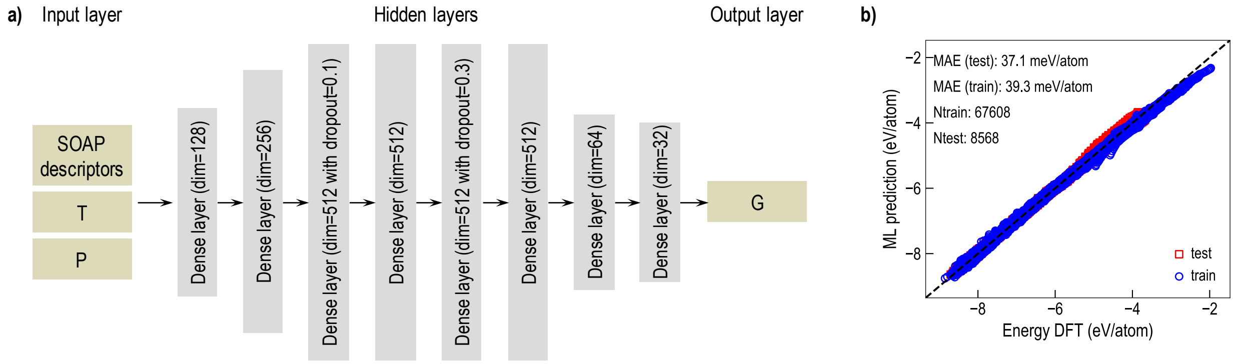

Supplementary Figure 3: a) Architecture of the DNN model used to learn Gibbs free energy and b) parity plot between the reference Gibbs free energy and respective DNN predictions for various phases of carbon in the training and the test set. The mean absolute error (MAE) in the DNN predictions for the training and test set are also included.

Using our surrogate ML model, we can quickly estimate the proximity of a newfound metastable phase with respect to the ground state, given only the structural information. The probability of realizing a metastable phase at a given temperature and pressure is directly proportional to $\exp(-\frac{\Delta G_{GS_i}^{MS_j}}{k_B T})$ with

$\Delta G_{GS_i}^{MS_j} = G_{MS_j} - G_{GS_i}$ where GS_i and MS_j are the ground state and the metastable phase of interest. Supplementary Figure 4 shows the predictions of the DNN at 31.25 GPa, $G(T, P = 31.25GPa)$, for metastable phases the ML model has never seen during training (S455, S291 and S389 are part of test set). The error between DFT and the ML predictions are less than 50 meV/atom (see Figure 6 in main manuscript). Thus, we can quickly classify a metastable phase as near-equilibrium and more likely to be synthesized, or far-from-equilibrium and less likely to be synthesized, by comparing free energies with ground state phases.

We also constructed a baseline ML model for performance comparison. The variation of free energy with temperature (T) and pressure (P) is observed to be somewhat quadratic in nature. A quadratic fit of the form, $G = c_0 + c_1^p P + c_1^t T + c_2^p P^2 + c_2^t T^2$ for *each individual phase*, resulted in an overall (train) mean absolute error (MAE) of ~13 meV/atom, suggesting that the free energy surface indeed varies quadratically with P and T. However, to make energy predictions for a new phase using such quadratic fit, the structural information of a new phase has to be mapped to the coefficients of the quadratic fit. Thus, we constructed another DNN model to predict the above P,T coefficients for a structure X, i.e., DNN(X) $\rightarrow (c_0, c_1^p, c_1^t, c_2^p, c_2)$, where X denotes the SOAP fingerprints of the new structure. As seen in the Supplementary Figure 5 the accuracy of this model (MAE of ~100 meV/atom) is significantly lower than that of the ML model developed in this work (MAE of ~36 meV/atom). This is mainly because including X, P, T together in the DNN model allows it to learn inter-dependencies of all the variables together.

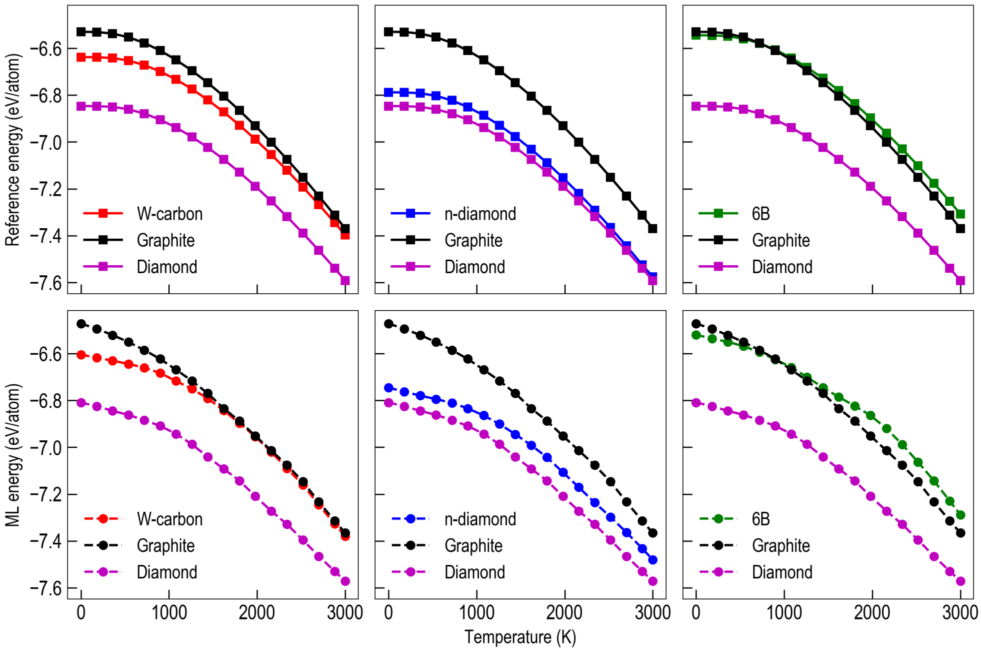

Supplementary Figure 4: (a),(b),(c) $G(T, P = 31.25$ GPa) computed using our workflow for W-carbon (S455), n-diamond (S291) & 6B (S389) phases, respectively. Cubic diamond and graphite are plotted alongside for comparison. (d),(e),(f) $G(T, P = 31.25$ GPa) computed using DNN for S455, n-diamond (S291) & 6B (S389) respectively. Cubic diamond and graphite are plotted alongside for comparison. The ordering of the different phases, along with free energy cross-over temperatures (see right most panels) are captured accurately.

1.4 Transformation barrier using crystal structure matching

While the metastable phase diagram provides information regarding the thermodynamics of the phases, an approximate kinetic barrier for transformation can be estimated for given pair of phases using the crystal structure mapping algorithm proposed by V. Stevanović et. al [18]. The algorithm determines the transformation between the unit cell and the optimal atom-to-atom matching which minimizes the Euclidean distance between the atoms and the number of chemical bonds broken during the transformation. Once a list of competing metastable phases are identified over a (T, P) of interest, we first map the crystal on to each other and generate linearly interpolated images across the transformation pathway determined by the algorithm. The transformation barrier is obtained by computing the energies of the generated images [18].

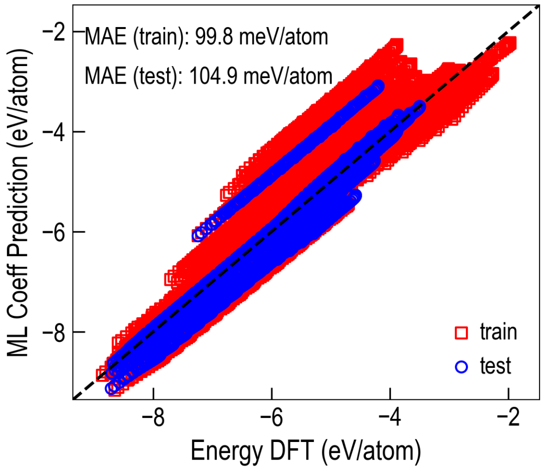

Supplementary Figure 5: Performance of the baseline DNN model fit to quadratic coefficients of P and T (see text for details), against the DFT data. This model shows poor performance compared to the DNN model developed in this work.

The structure matching algorithm and the image generation is implemented within the PYLADA software package [19]. As an example, the transformation barriers for metastable bct-C and Z-carbon phases, starting from ground state cubic-diamond and graphite, are shown in Supplementary Figure 6.

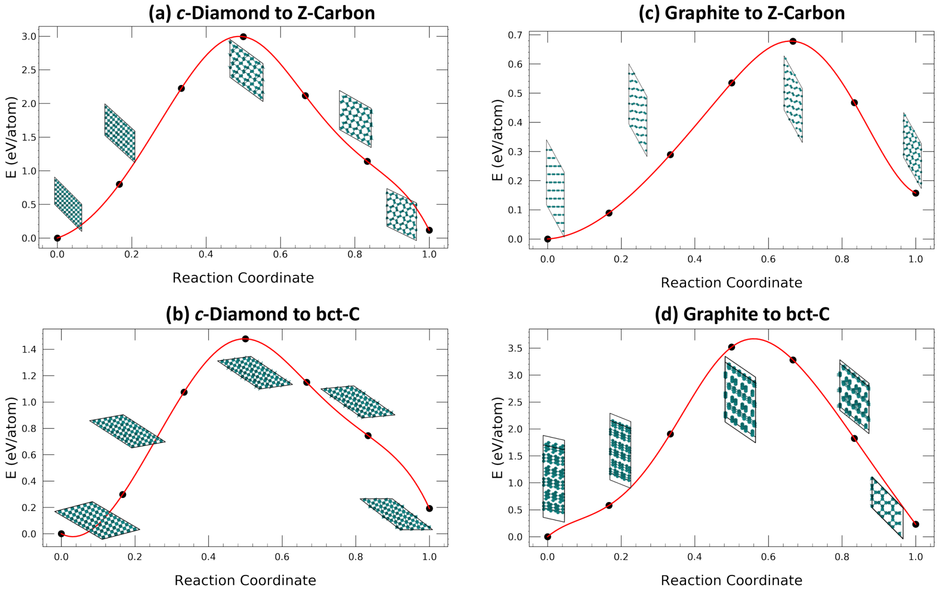

Supplementary Figure 6: Energy barriers for (a) cubic-diamond to Z-carbon, (b) cubic-diamond to bct-Carbon, (c) graphite to Z-carbon and (d) Graphite to bct-Carbon, by matching the phases on to each other and computing the energies of the images along the transformation pathway.

1.5 Solid state Nudged Elastic Band (SSNEB) calculations

The initial conditions for the images were obtained by matching the crystal structures of the competing phases with each other ([18]). We then perform SSNEB with climbing nudged elastic band algorithm as implemented in VTST package. The spring constant used in our simulations is -4.5 $eV/\AA^2$. All calculations were performed in VASP. Fig 7 compares the kinetic barriers corresponding to graphite to Z-carbon and graphite to hexagonal diamond transformations.

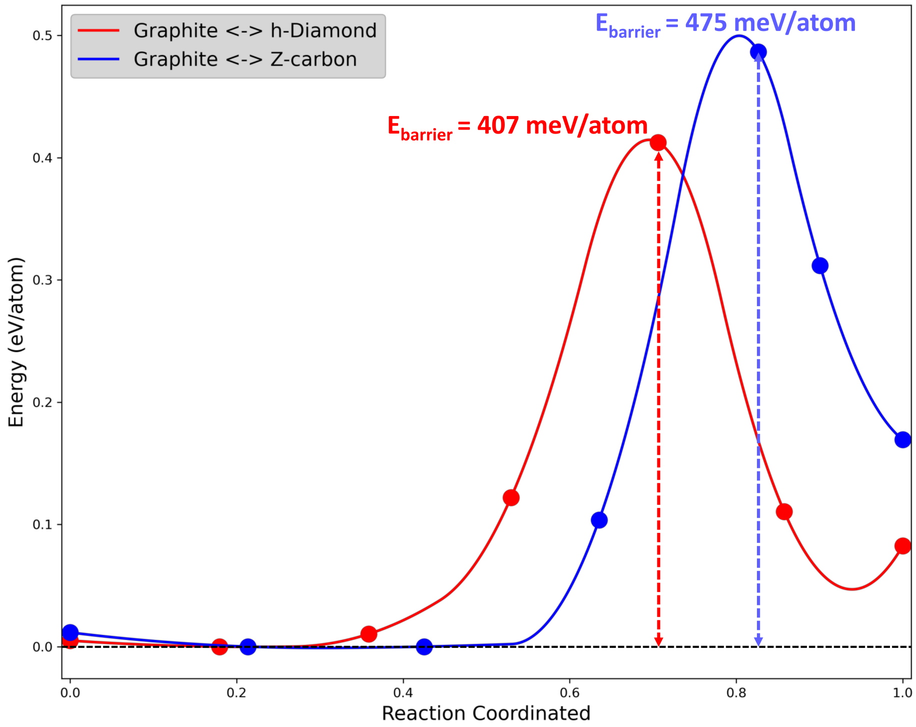

Supplementary Figure 7: Kinetic barrier along the minumum energy pathway computing using solid state nudged elastic band (SSNEB) calculations at $P = 0GPa$ for graphite to Z-carbon (blue) and graphite to hexagonal diamond (red) transformations

1.6 Transmission Electron Microscopy

In this work, after decompression from high pressure and temperature treatment, we opened the DACs, transferred the samples from the chamber to a clean marble mortar with a tiny pin. TEM samples were prepared by crushing the recovered sample using a marble mortar and pestle and then dispersing these crushed powders onto a holey carbon grid. Focused-ion beam (FIB) technique is also used to prepare plane-view and cross-sectional TEM specimens. Argonne Chromatic Aberration-corrected TEM (ACAT, FEI Titan 80-300ST TEM/STEM) with a field-emission gun was used to investigate the crystallographic orientation, high-resolution transmission electron microscopy (HRTEM) images from the recovered samples.

2 Supplementary Discussion

2.1 Stability of stacking disorder diamond phases

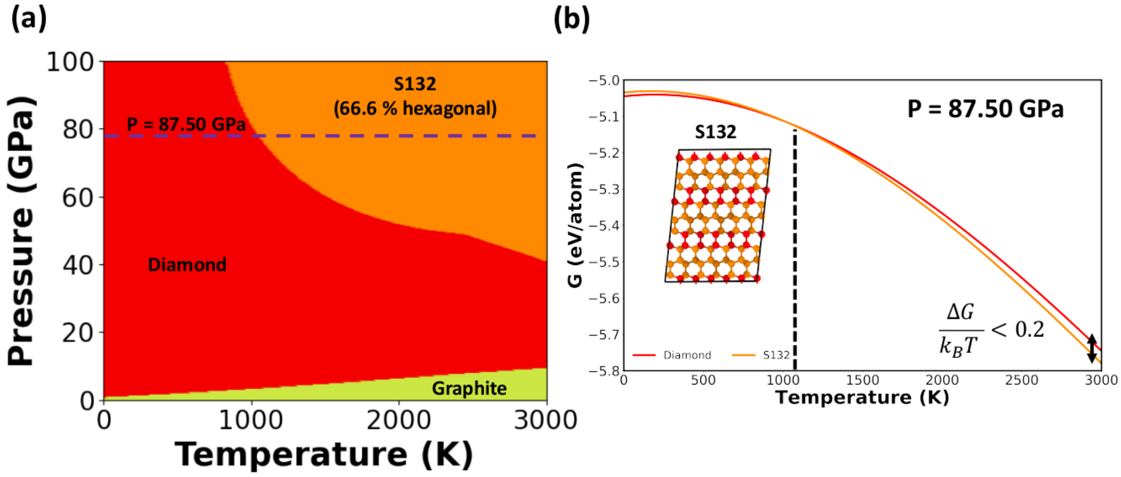

Supplementary Figure 8: Equilibrium phase diagram including the stacking disorder diamond phase S132

Supplementary Figure 8 (a) shows the equilibrium phase diagram constructed by comparing the $G(T, P)$ of *all* the candidate phases identified by our algorithm. We note that the stacking disorder diamond phase (S132) is marginally stable ($\Delta G/k_BT < 0.2$, see Fig 8) compared to cubic-diamond over the high-pressure-high-temperature region. While pure cubic-diamond has an "ABCABC" stacking pattern, stacking disorder phases can be regarded as a mixture of "ABAB" hexagonal-stacking and ABCABC cubic-stacking. The structure of S132 is shown in Supplementary Figure 8(b). The red and orange atoms correspond to atoms with cubic and hexagonal like co-ordination environment. Observations of stacking disorder phase with varying content of hexagonal diamond under such conditions are well known and reported in the past[20–32].

We further inspect the ΔG between stacking disorder (S132) and cubic diamond. Supplementary Figure 8(b) shows the Gibbs free energy profile at P=87.50 GPa, indicated as purple dashed line in Supplementary Figure 8(a). The maximum difference with respect to the stable phase are at 3000 K with $\Delta G^{S132}_{Diamond}(T = 3000K, P = 37.5GPa) = -35$ meV/atom. At 3000 K, $\Delta G/k_BT < 0.2$ suggests a very high probability of stacking disorder diamond co-existing with cubic-diamond in agreement with experimental observations [20–32]. Our results suggests that such stacking faults are marginally stabilized at high temperatures due to entropic contributions.

2.2 Comparison of LCBOP and DFT equation of states

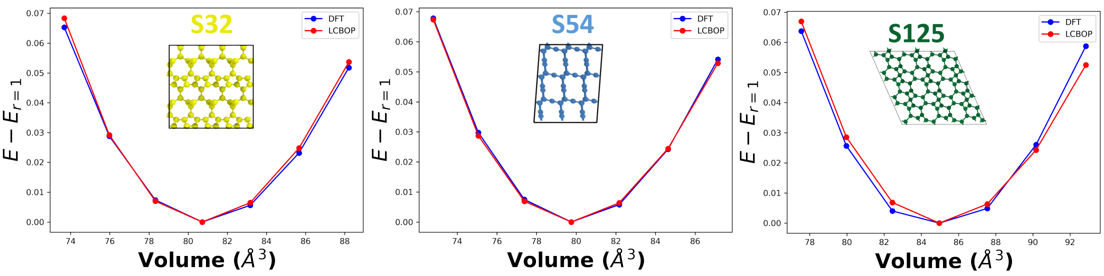

Supplementary Figure 9: Equation of state computed using DFT and LCBOP potential. The energy at the original volume (or volume ratio, $r = 1.0$) is taken to be the reference for both LCBOP and DFT

Here, we benchmark the accuracy of LCBOP potential for the metastable phases , for which the dynamical stability were inspected, by comparing the equation of state, $E(V)$, with DFT. We scale the unit cells of metastable phase with volume ratio, $r \in (0.95, 1.05)$, and compute the energy of the scaled system to obtain

the $E(V)$. While the energy of the isolated atom in the classical LCBOP model is zero, DFT include the electronic energies of the atoms. This difference in the reference values results in a constant energy offset between the two equation of states. To make a fair comparison, we compare the $E(V) - E_{r=1.0}$ curves of both DFT and LCBOP model. The LCBOP predicted equation of states matches excellently with DFT (Supplementary Figure 9).

2.3 *n*-diamond

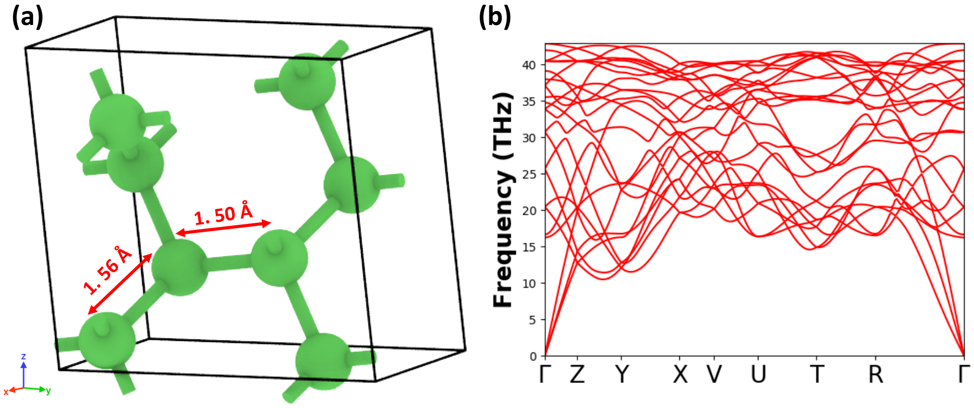

Supplementary Figure 10: (a): Structure of *n*-diamond after relaxation, (b) phonon dispersion of *n*-diamond

The initial structure of *n*-diamond (S291) as identified by our evolutionary algorithm is relaxed under an anisotropic pressure of 48 GPa in the y-direction and 20 GPa in the x- and z- directions. The resulting structure is still a cubic diamond like structure with two different bond lengths of 1.56 Å and 1.50 Å (Supplementary Figure 10). The simulated diffraction pattern of the final structure matches well with the previously reported *n*-diamond structure. We next inspect the stability of the proposed structure by computing the phonon spectrum and checking for any possible imaginary modes. The phonon spectrum is computed using PHONOPY package [33] with force constants obtained form density functional perturbation theory (DFPT). The relevant high symmetry points labeled in the phonon spectrum were obtained using the algorithm described in Ref.[34] which uses the spglib library [35] to construct the brillouin zone. The structure is stable since there are no imaginary modes.

Cubic-diamond consists of two fcc lattices that shift along [111] diagonal direction respect to each other. When the shift distance equals to a sp^3 bond length, (200) diffraction spots extinguish since cubic-diamond has one sp^3 bond length. When the shift distance of these two fcc lattices is away from 1.54 Å, the intensity at (200) diffraction spots gradually increases. In the simulated diffraction pattern using S291 structure, we can find the intensity of (200) spots are much lower than (220) spots due the small difference in these two bond lengths (1.56 Å, 1.50 Å). In the experiment diffraction pattern, the (200) intensity is close to the (220), indicating this n-diamond has a much smaller bond length than 1.50A.

2.4 Performance comparison of machine learning methods

Previous studies from other fields such as computer vision, suggest that for problems that involve extremely large volume of data (thousands to hundred thousands of points), deep learning (DL) techniques perform superior to the other ML models. To further validate this point for the current problem of learning free energy, we developed Gaussian process regression (GPR) models using similar training methodology. Since training GPR models become prohibitive when no. of training data points is large, we restricted no. of training data points to a maximum value of 5000. The best GPR model had a test error of 68 meV/atom as compared to 37 meV/atom for the case of DL model (see Supplementary Figure 11). We emphasize that in this work we decided to use DL methods for learning free energy because of the large volume of available training data, and prior work in the general field of ML as well as ML-based force fields, showcasing the ability of DL techniques to outperform relatively less complex methods such as linear regression.

2.5 Diaphite-like lonsdaelite phase

Evidence of diaphite-like lonsdaelite phase are provided in Ref [36]. Specifically, Figure 2 of Ref [36] shows the AC-HRTEM image of lonsdaleite along $[11\bar{2}0]$ with two different bond lengths ($OA \approx 1.56$ Å and $OB \approx 1.47$

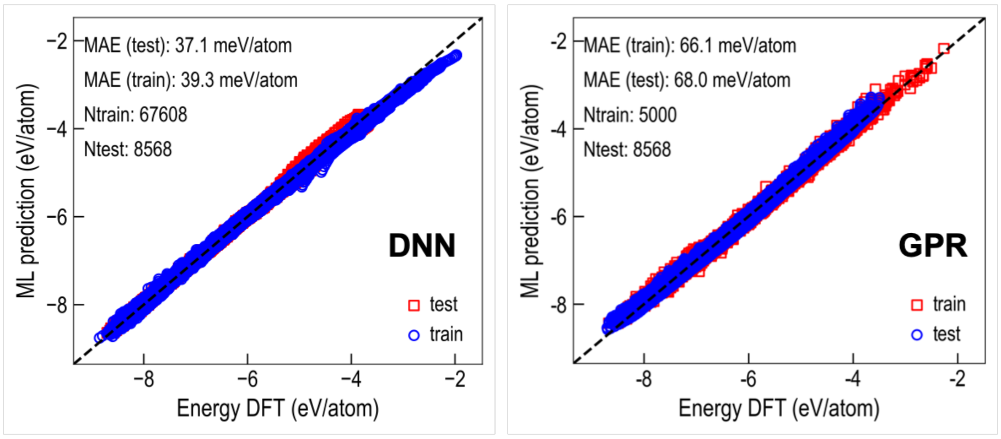

Supplementary Figure 11: Performance of DNN model utilized in this work against Gaussian process regression (GPR) model with exactly same test dataset.

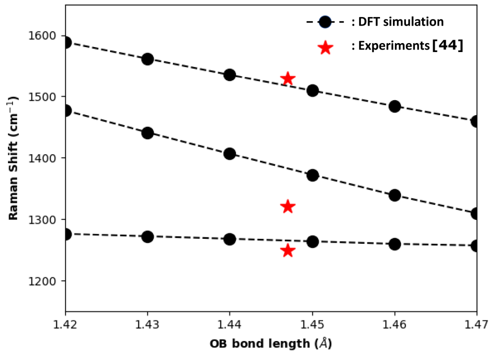

Supplementary Figure 12: Raman frequencies computed from DFT as a function of bond length OB with fixed bond length OA (1.56 Å). Experimentally observed Raman vibration frequencies [36] are indicated by red stars

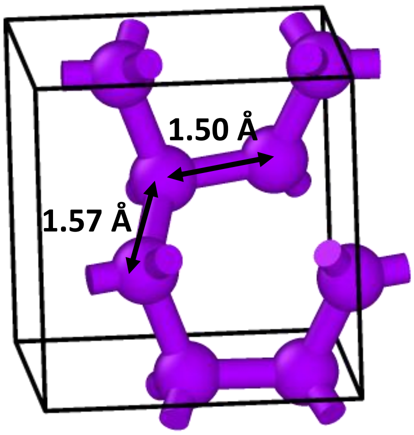

Supplementary Figure 13: Diaphite-like lonsdaelite phase [36] (S353) with two different bond length and metastable at $\Delta G = 140\ meV/atom$

Å) and Lonsdaelite phase after relaxing under anisotropic pressure. Figure 12 shows the DFT calculated Raman frequency dependence on the OB bond length while keep the OA bond length fixed at 1.56 $\AA$. The red stars indicate the experimental Raman frequencies from Ref [36].

3 Supplementary Figures

3.1 Metastable Phase Diagrams with only the phases identified in evolutionary search

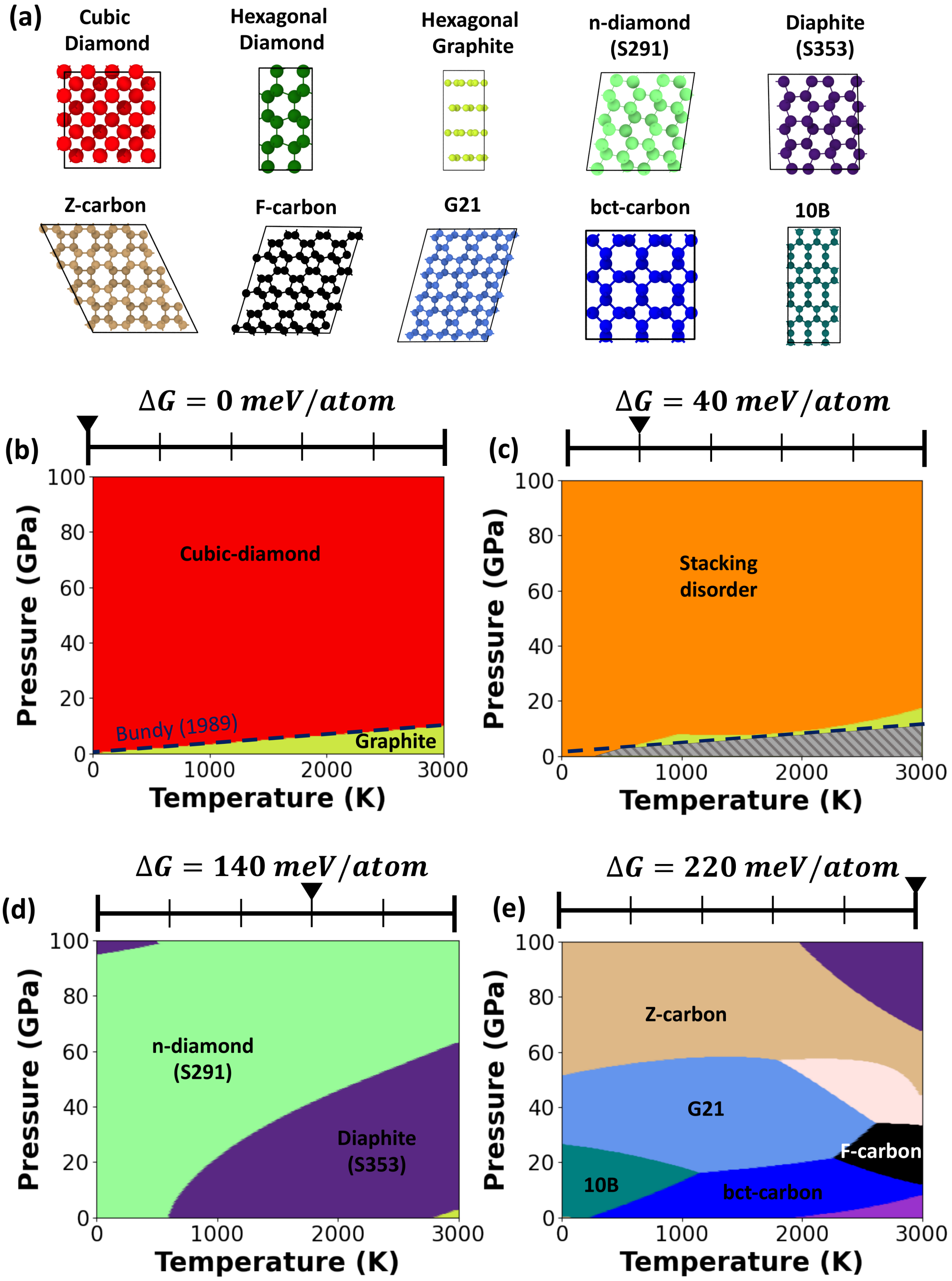

Supplementary Figure 14: (a) Near equilibrium phases identified by evolutionary structure search; (b) equilibrium phase diagram with boundary fitted using MSVM. Equilibrium phase diagrams matches with the experimental phase diagram[4, 37]; (c),(d) & (e) metastable phase diagram (at a ΔG of 40, 140 and 220 meV/atom respectively) showing metastability of phases listed above. Regions where no metastable phase is present other than the ground state are shaded in grey

3.2 Relative stability of far from equilibrium structures

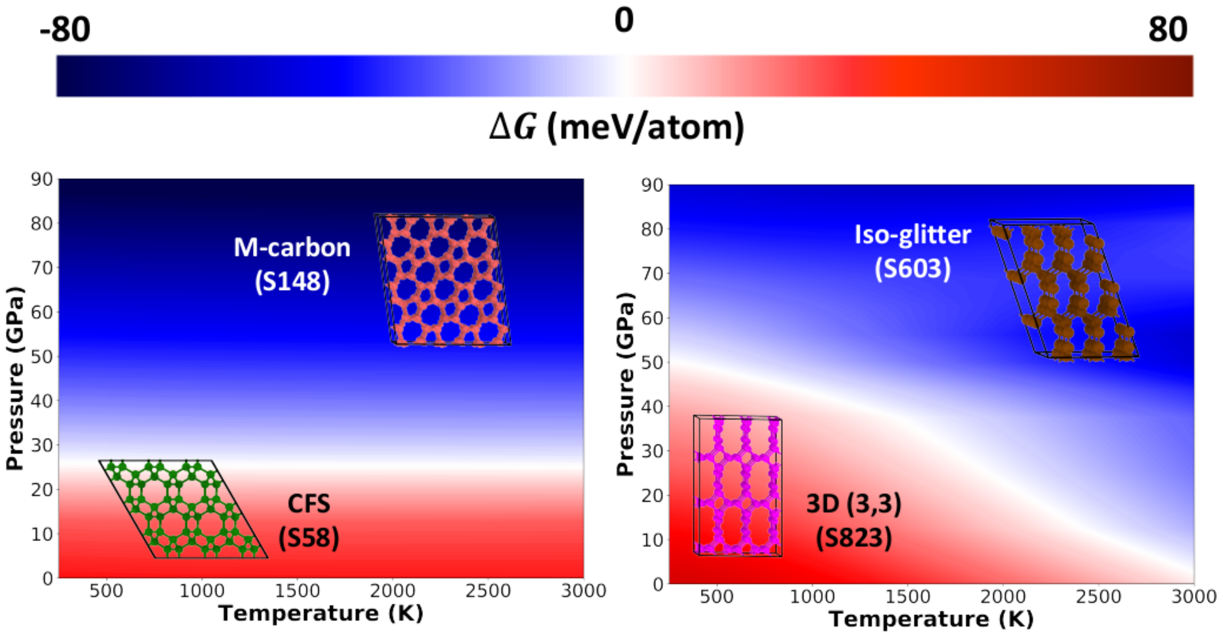

Supplementary Figure 15: Relative stability of far from equilibrium structures

3.3 Transformation barriers across metstable phases

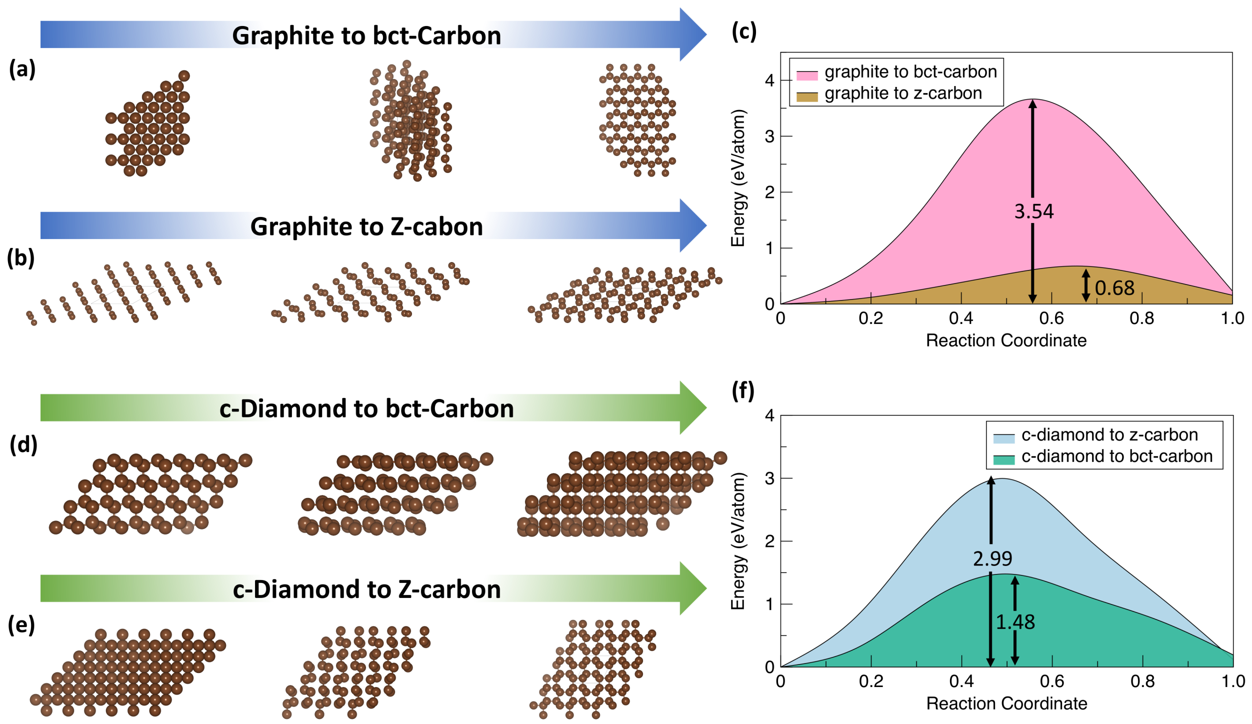

Supplementary Figure 16: The transformation barriers ((c),(f)) are obtained by matching the crystal structure onto each other and computing the energies of the linearly interpolated images across the transformation pathway. The geometries of the initial, intermediate and final images for graphite to bct-carbon, graphite to Z-carbon, c-diamond to bct-carbon, and c-diamond to Z-carbon, are shown in panel (a),(b),(d)&(e) respectively

3.4 Far from equilibrium metastable phase diagrams

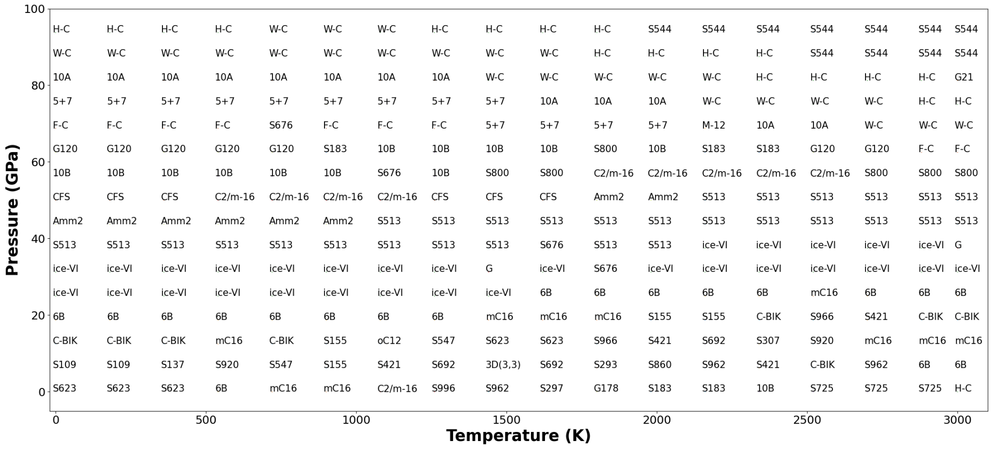

(a) $\Delta G = 300 meV/atom$

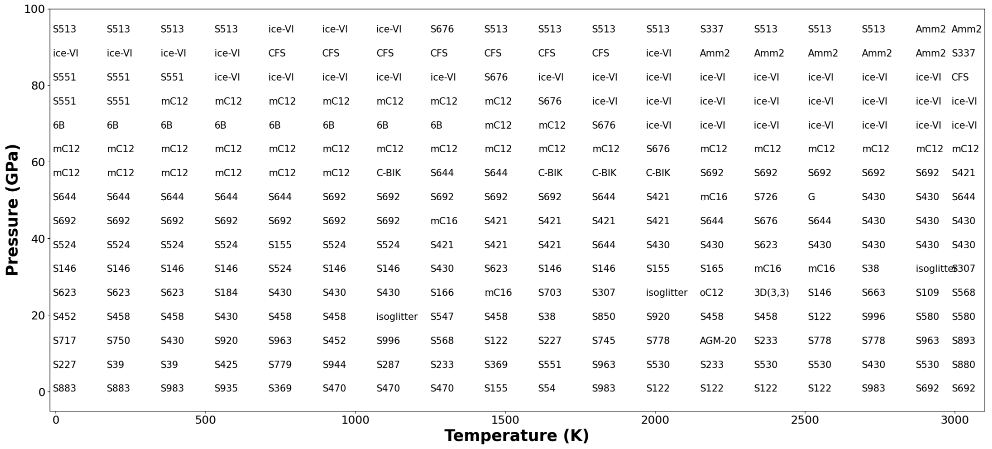

(b) $\Delta G = 400 meV/atom$

Supplementary Figure 17: Far-from-equilibrium metastable phase diagram

3.5 Metastable phase diagrams of select few phases

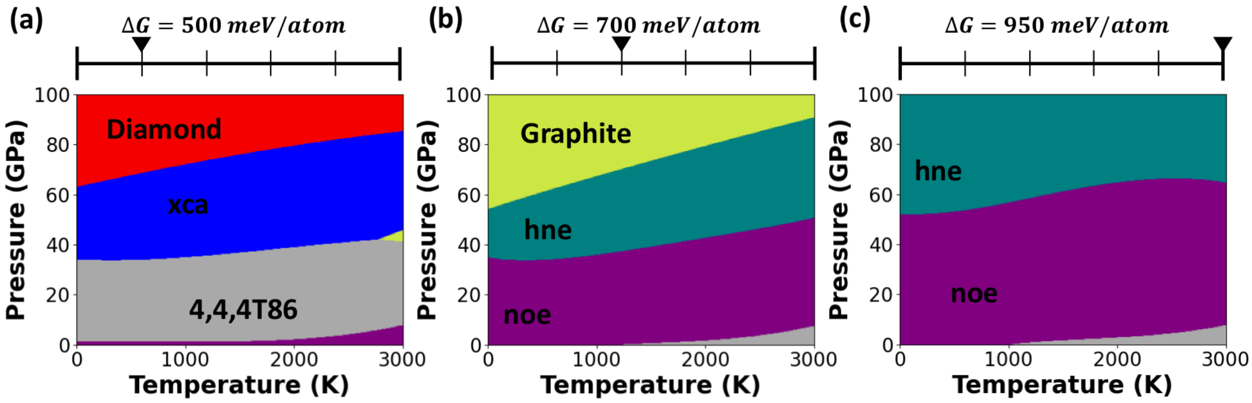

Supplementary Figure 18: Metastable phase diagram only considering S134 (hne), S421 (xca), S419 (4,4,4T86), S30 (noe), diamond and graphite at (a) ΔG=500 meV/atom, (b) ΔG=700 meV/atom and (c) ΔG=950 meV/atom

4 Supplementary Tables

4.1 Phases already reported in SACADA

Table **Supplementary Table 1**: List of metastable phases within $\Delta H_{cut-off} = 670 meV/atom$ matching with structures from SACADA

Structure Index	Reported Names	Space group
S224	3D-(5,0); mC16-carbon	Cmcm
S389	6B	C12/m1
S400	mC12	A12/n1
S603	isoglitter	Cmmm
S488	C-BIK	Cmcm
S896	G158	Imma
S112	10A	C12/m1
S375	H-6;6(3)6-09	P6222
S111	F-carbon; Z-carbon-1; J-carbon; S-carbon; M10-carbon; F-C	P12/m1
S148	"5+7"; M-carbon; M-12	C12/m1
S160	ZGM-12; oC12-carbon; IGN	Cmcm
S435	Z-carbon-3; 12C	C12/m1
S58	CFS; unj; 6A; P6522	P6122
S0	Structure I; C1(41); 10^3; 4(3)1; K4; srs	I4132
S159	(RL)2; oC16-II; Cco-C8; Z-carbon; oCco-C8; C8-carbon; 3D(2,2)-III; sie; Cmmm	Cmmm
S19	Diamond; LA1	Fd-3m
S86	12R	R-3m
S677	(3,0)/(4,0); 10B	Cmmm
S127	G21	P121/m1
S60	A Hypothetical Metallic-Allotrope of Carbon; bct-4; sp2 allotrope; Hinged; poly-acetylene; 4(3)2; Bct-C4	I41/amd

S513	8-tetra(2,2)-tubulane; (R2), bct-C4; Rectangulated carbon; D; 3D(2,2)-II; bct-carbon; crb; 3D-(2,2); LA3; bct4-carbon; C4	I4/mmm
S596	mC16-carbon	C12/m1
S319	cfc; 4H-diamond	P63/mmc
S41	6(3)1-10; Rh6	R-3m
S823	3D (3,3)	Cmmm
S40	bco-C16	Imma

4.2 Know topological nets not present in SACADA

Supplementary Table 2: Structures with topological nets not present within SACADA database [38]. The topological nets are characterized by the ToposPro software package [39]

Structure Index	$E - E_{dia}(eV)$	Topology	Net Type	Point Symbol of Net
S134	0.208	hne	3,3,3-c	{5.7^2}3{5^2.7}
S421	0.286	xca	3,4-c	{6^3}{6^6}2
S419	0.354	4,4,4T86	4,4,4-c	{4.5^2.6^3}{5^3.6^3}
S30	0.383	noe	3,3,3-c	{7.12^2}{7^2.8}3
S38	0.435	nof	3,3-c	{6.10^2}{6^2.10}
S235	0.675	3,3,3,3T2	3,3,3,3-c	{11^3}{5.11^2}5
S338	1.571	cem-a	3,3,3-c	{5.6.8}2{5.6^2}2{5.8^2}

4.3 New topological Nets

Supplementary Table 3: Structures with topological nets not present within Topological Types Database (TTD) [40, 41] as identified by the ToposPro software package [39]

Index	$E - E_{dia}(eV)$	Net Type	Point Symbol of Net
S83	0.135	3,3,3,4-c	{6^3}3{6^5.8}
S112	0.172	4,4,4,4,4-c	{5^2.6^3.8}{5^2.6^4}2{5^3.6^3}{6^5.8}
S260	0.228	3,3,3,3,4-c	{6^3}4{6^5.8}
S623	0.244	3,3,4-c	{4.6^3.8^2}2{6^3}3
S297	0.276	3,3-c	{5.8^2}{5^2.8}2
S487	0.283	3,3,3,3,4,4-c	{5.6^3.7^2}{5.7^2}2{5^2.6^2.7^2}{6^3}2
S596	0.290	3,4-c	{4.6^3.8^2}{6^3}
S108	0.303	3,3,3,3-c	{5.12^2}2{5.6.9}2{5.6^2}{6^2.10}
S530	0.318	3,3,3,3,4,4-c	{5.6^3.7^2}2{5.7^2}{5^2.7}{6^3}2
S424	0.322	3,4,4,4,4-c	{5.7^2}2{5^3.7^3}2{5^4.7.8}{5^4.7^2}2{5^5.7}
S54	0.334	3,3,3,4,4-c	{5.6^3.7.8}{5^2.6^2.7.8}{5^2.8}{6^3}2
S470	0.337	3,3,4-c	{6^3}3{6^5.8}2
S568	0.352	3,3,4,4-c	{5.6^3.7^2}2{5.7^2}{5^2.7}
S698	0.379	3,3,4,4-c	{4.5.7^3.8}2{5.7^2}{5^2.7}
S529	0.406	3,3,4,4-c	{5.6^3.7.8}{5^2.6^2.7.8}{5^2.8}{6^3}
S763	0.408	3,4,4,4,4-c	{5.6.8^4}{5.6.8}2{5.6^4.8}{5^2.6^3.8}2{5^3.6^3}2

Continued on next page

Index	$E - E_{dia}(eV)$	Net Type	Point Symbol of Net
S907	0.410	3,3,3,3,3,3,4,4,4,4-c	{4.5.6.7^2.8}{4.6.7^2.8^2}{5.6.7}{5.7^2}{5^2.7}
S857	0.412	3,4,4,4-c	{4.5.6^3.8}2{5.6^2}{5.6^5}2{6^6}2
S912	0.418	3,3,4,4,4,4,4,4,4,4,4,4-c	{4.5.6^3.7}{4.5^3.6.7}3{5.6.7}{5.6^3.7^2}{5.6^...
S310	0.424	3,3,3-c	{5.12^2}3{5^2.8}
S47	0.427	3,4-c	{4^2.6^2.8^2}{6^2.8}
S589	0.434	3,3,3,4,4-c	{4.6.8}{4.6^3.8^2}{4^2.6^2.8^2}{6^2.8}2
S234	0.438	3,3,3,3,3,3,3,3,3,3-c	{4.5.7}2{4.7.9}2{5.7.9}4{5^2.7}{5^2.8}
S866	0.455	3,3,4,4,4,4,4,4,4,4-c	{5.6.7}2{5.6^3.7^2}{5.6^4.7}{5.6^5}2{5^2.6^2.7...
S358	0.456	3,3,4-c	{6^2.10}3{6^3.10^3}
S994	0.475	3,4,4-c	{5.6^3.7.8}2{5.8^2}{5^2.6^4}
S999	0.487	3,3,4,4-c	{4.6.8}{4.6^3.8^2}{6^3}{6^5.8}
S455	0.489	3,4-c	{5.8^2}{5^2.6^2.7.8}2
S70	0.501	3,3,3,3,3,3,3,3-c	{5.10^2}{5.7.10}4{7.10^2}3
S26	0.506	3,3,3-c	{6.8^2}3{8.12^2}
S336	0.509	3,3,3,3,3,3,3,3,3,3,4-c	{5.6.9.12^2.13}{5.6.9}3{5.6^2}{6.12^2}{6^2.9}4...
S856	0.520	3,3,4,4-c	{5.6^3.7^2}{5.7^2}2{5^2.6^2.7.8}
S397	0.525	3,3,3,3,3,3,3,3,3,3,4-c	{5.6.9.13^3}{5.6.9}3{5.6^2}{6.13^2}{6^2.9}4{6^3}
S964	0.538	3,4,4-c	{3.6^3.7^2}4{3^2.10^4}{6^3}2
S407	0.538	3,3,3,3,4-c	{5.6.9}2{5.6^2}2{5.9^4.12}{6.9^2}2{6^2.9}2
S892	0.549	2,3,3,3-c	{16}{5.12^2}2{5.16^2}{5^2.8}
S342	0.566	3,3,4,4,4,4-c	{3.5.6.7.8.9}2{3.5.8^2.9^2}2{3^2.9^4}{5.6.8}2{...
S688	0.567	3,3,3,3,3,3,4,4,4,4-c	{5.7.8}2{5.8^3.9^2}{5^2.7.8^2.9}{5^2.7}{5^2.8}...
S133	0.580	3,3,3,3,3-c	{5.8.9}2{5.8^2}{5.9^2}2{8.9^2}2{8^2.9}
S121	0.588	3,3,3,3,3,3,3,3,3,3,4-c	{5.6.7.9.10^2}{5.6.7}{5.7.10}{5.7.8}{5.8.10}{6...
S830	0.595	3,3,3,3,3,3,3,3,3,3,4-c	{4.6.8}2{4.7.9.12^2.13}{4.7.9}{6.7.9}2{6^2.7}2...
S711	0.599	3,3,3,4,4-c	{5.6^4.9}{5.9^2}{5^2.6^3.9}2{5^2.8}2{6.9^2}
S701	0.601	2,3,3,3,3,3,3,4-c	{10.12^2}{12}{5.10^2}{5.12^2}{5^2.8.10^2.11}{5...
S114	0.609	3,3,3,3,3,3-c	{5.9^2}3{5^2.8}{9.12^2}
S165	0.625	3,3-c	{8.10^2}
S941	0.632	3,3,4-c	{4.8^2}{4.8^3.10^2}{8^3}2
S869	0.643	3,3,3,3,4-c	{10^3}{5.10^2}{5.10^4.12}{5^2.8}4
S773	0.653	4,4,4,4,4,4,4,4,4,4-c	{3.4.5.6.7.8}{3.4.5^2.6.7}{3.4.5^2.6^2}{3.4.6....
S210	0.656	3,3,3,3,4-c	{5.6.9}2{5.8.11}2{5.8^2.11^3}{6.8.11}4
S152	0.678	3,4,4-c	{5.10^2}2{5^4.8^2}{5^5.8}4
S192	0.698	3,3,3,3,3,3,4,4,4-c	{4.6.8^3.10}{4.6.8}{4.6^2.8^3}2{6.8^2}2{6^2.10...
S748	0.699	3,3,3,3,4,4,4,4,4-c	{6.7^2}3{6^2.7}{6^3.7^3}{6^4.7.8}2{6^5.8}{6^6}
S610	0.706	3,3,4,4,4,4,4-c	{3.5^2.8^2.9}{3.5^3.6.8}{3^2.4.5.6.7}{3^2.4.9^...
S976	0.712	3,3,3,3,3,3,4-c	{5.6.9^3.10}{5.6.9}3{5.9^2}{6.9^2}2
S315	0.730	3,3,3,3,3,3,4-c	{6.7^2.11^3}{6.7^2}5{7.11^2}
S578	0.732	3,3,3,3,4-c	{5.6.7}2{5.6.8}2{5.7.8^2.11^2}{6.7.8}2{7.12^2}2
S542	0.733	3,3,3,3,4,4,4-c	{3.6.7^3.8}2{3.7.10^4}{6.7^2}4
S528	0.755	3,3,3,3,4,4,4-c	{4.10^2}{4.5.8^2.9.10}{4.5^2.7^2.8}2{5.10^2}{5...
S956	0.755	3,3,3,3,3,3,4-c	{5.6.9.10^3}{5.6.9}3{5.9^2}{6.10^2}{6.9^2}
S51	0.755	3,3,3,3-c	{7.10.11}{7.10^2}{7^2.10}{7^2.8}
S78	0.757	3,3,3,3,3,3,3,3-c	{6.11^2}{6.7.9}4{6.9^2}{7.11^2}{7.9^2}

Continued on next page

Index	$E - E_{dia}(eV)$	Net Type	Point Symbol of Net
S849	0.770	3,3,3,3,4,4,4-c	{3.6.7.8^2.9}{3.6.7.9^3}{3.6.8^2.9^2}{6.8^2}3{...
S45	0.775	3,3,3,3,3-c	{7.10^2}{7.9.10}2{7.9^2}4{9.10^2}
S826	0.776	3,3,3,4-c	{4.6.8^3.10}{4.6.8}{6.8^2}{8^3}
S416	0.786	2,3,3,3,3-c	{12}{6.10^2}4{6.12^2}2
S835	0.827	3,3,3,3,3,3,4-c	{6.11^2}{6.7^2}4{6.7^3.10.11}{7.11^2}
S700	0.828	2,3,3,3,3-c	{11^3}{13}{5.11^2}3{5.13^2}2
S724	0.829	3,3,3,3,3,3,3,3,4,4,4-c	{3.7.8^3.9}{3.7.8^4}2{7.8^2}4{8.10^2}2{8^3}2
S524	0.831	3,3,4,4,4,4,4,4,4,4-c	{6^3}{6^6}4
S573	0.841	3,3,3,3,3,3,4-c	{6.10^2}2{6.8^2}{6^2.8^3.10}{6^2.8}3
S791	0.859	3,3,4,4-c	{3.6.7.8^3}{3.6.7}{3.8^3.9^2}{6.8^2}
S922	0.862	3,3,3,3,4,4,4,4-c	{3.5.7^2.9^2}{3.7^3.8^2}2{5.7.9}3{5.7^4.9}{7^3}
S423	0.863	3,3,3,3,4-c	{6.8^2}2{6^2.8^4}{6^2.8}
S867	0.946	3,3,3-c	{4.6.8}2{6^2.8}2{6^3}
S434	0.971	2,3,3,3,3-c	{9^3}6{9}
S425	0.982	3,3,3-c	{5.6.8}2{5.8^2}{5^2.6}
S660	0.993	3,3,3,3,3,3,3,3-c	{6.11.12}{6.11^2}{6.7.9}4{7.9^2}2
S232	1.048	3,4,4-c	{3.6^3.7^2}4{3^2.10^4}{6^3}4
S674	1.193	2,3,3-c	{12}{5.12^2}{5^2.8}2
S842	1.240	3,3,3,3,3,3,4,4,4-c	{5.10.11}{5.10^2}{5^2.8}4{5^3.8^3}2{5^4.8^2}
S395	1.243	3,3,3,3,3,3,3,3,4,4,4-c	{5.7.8}{5.7.9}{5.7^2}2{5^2.7.8^2.10}2{5^2.7}2{...
S621	1.248	3,3,3,4-c	{5.6.9}4{5.9^4.12}{6.9^2}2
S787	1.271	3,3,3,3,3,3,4-c	{10^3}{5.10^2}2{5^2.8.10^2.11}{5^2.8}3
S184	1.324	3,3,3,4,4-c	{6^3}3{6^5.8}2
S664	1.363	3,3,3,3,4,4,4-c	{4.5.7.8^3}{4.5^2.7.8^2}{4.5^2.8^2.9}{4.8^2}{5...
S903	1.382	3,3,3,3,3,3,4-c	{10^3}{4.6.8.10^2.12}{4.6.8}3{6.10^2}2
S768	1.392	3,3,4,4,4,4,4-c	{3.4.5^2.6.8}2{3.4^2.8^2.9}{4.5.7}{4.8^4.10}{4...
S109	1.457	3,3,3,3,3,3-c	{5.6.7}2{5.7^2}{5^2.7}{6^2.7}2
S158	1.746	3,3,3,3,3-c	{11^2.12}{5.11^2}{5.6.9}4{6.11^2}2
S740	1.864	3,3,3,4,4-c	{3.5^2.6.8.9}2{3.9^4.10}{5.9^2}2{5^2.8}2
S747	2.040	3,3,4,4,4,4,4,4,4-c	{5^2.6.8^3}{5^2.6^3.8}{5^3.6^3}2{5^4.6.8}2{6.8...
S808	2.384	3,3,4,4,4,4,4,4,4-c	{6^2.8^4}{6^2.8}2{6^5.8}2{6^6}4
S534	2.826	2,3,3,3-c	{7.11^2}6{7}
S944	3.252	3,3-c	{4.6.8}{6^2.8}
S452	4.053	3,3,4,4-c	{4.6^5}{6^3}
S868	4.813	2,3,3,3,3-c	{10^3}3{10}

Supplementary References

[1] J. c. v. Klimeš, D. R. Bowler, and A. Michaelides, "Van der waals density functionals applied to solids," *Phys. Rev. B*, vol. 83, p. 195131, May 2011. [Online]. Available: https://link.aps.org/doi/10.1103/PhysRevB.83.195131

[2] M. Dion, H. Rydberg, E. Schröder, D. C. Langreth, and B. I. Lundqvist, "Van der waals density functional for general geometries," *Phys. Rev. Lett.*, vol. 92, p. 246401, Jun 2004. [Online]. Available: https://link.aps.org/doi/10.1103/PhysRevLett.92.246401

[3] G. Román-Pérez and J. M. Soler, "Efficient implementation of a van der waals density functional: Application to double-wall carbon nanotubes," *Phys. Rev. Lett.*, vol. 103, p. 096102, Aug 2009. [Online]. Available: https://link.aps.org/doi/10.1103/PhysRevLett.103.096102

[4] L. M. Ghiringhelli, J. H. Los, E. J. Meijer, A. Fasolino, and D. Frenkel, "Modeling the phase diagram of carbon," *Physical Review Letters*, vol. 94, no. 14, p. 145701, 2005. [Online]. Available: https://link.aps.org/doi/10.1103/PhysRevLett.94.145701

[5] G. Kresse and J. Furthmüller, "Efficient iterative schemes for ab initio total-energy calculations using a plane-wave basis set," *Phys. Rev. B*, vol. 54, pp. 11 169–11 186, Oct 1996. [Online]. Available: https://link.aps.org/doi/10.1103/PhysRevB.54.11169

[6] S. Plimpton, "Fast parallel algorithms for short-range molecular dynamics," *Journal of Computational Physics*, vol. 117, no. 1, pp. 1–19, 1995. [Online]. Available: http://www.sciencedirect.com/science/article/pii/S002199918571039X

[7] W. W. T. B. C. Revard and R. G. Hennig, "Genetic algorithm for structure and phase prediction," https://github.com/henniggroup/GASP-python, 2018. [Online]. Available: https://github.com/henniggroup/GASP-python

[8] B. C. Revard, W. W. Tipton, and R. G. Hennig, *Structure and Stability Prediction of Compounds with Evolutionary Algorithms*. Cham: Springer International Publishing, 2014, pp. 181–222. [Online]. Available: https://doi.org/10.1007/128_2013_489

[9] W. K. Tipton and R. G. Hennig, "Gasp: The genetic algorithm for structure and phase prediction," 2014.

[10] C. Cortes and V. Vapnik, "Support-vector networks," *Mach. Learn.*, vol. 20, no. 3, pp. 273–297, 1995.

[11] C. J. Burges, "A tutorial on support vector machines for pattern recognition," *Data Mining and Knowledge Discovery*, vol. 2, no. 2, pp. 121–167, 1998. [Online]. Available: https://doi.org/10.1023/A:1009715923555

[12] E. J. Bredensteiner and K. P. Bennett, "Multicategory classification by support vector machines," *Computational Optimization and Applications*, vol. 12, no. 1, pp. 53–79, 1999. [Online]. Available: https://doi.org/10.1023/A:1008663629662

[13] Z. Wang and X. Xue, *Multi-Class Support Vector Machine*. Cham: Springer International Publishing, 2014, pp. 23–48. [Online]. Available: https://doi.org/10.1007/978-3-319-02300-7_2

[14] K. Crammer and Y. Singer, "On the algorithmic implementation of multiclass kernel-based vector machines," *J. Mach. Learn. Res.*, vol. 2, pp. 265–292, 2002.

[15] Y. Lee, Y. Lin, and G. Wahba, "Multicategory support vector machines," *Journal of the American Statistical Association*, vol. 99, no. 465, pp. 67–81, 2004. [Online]. Available: https://doi.org/10.1198/016214504000000098

[16] Y. Guermeur and E. Monfrini, "A quadratic loss multi-class svm for which a radius-margin bound applies," *Informatica, Lith. Acad. Sci.*, vol. 22, pp. 73–96, 2011.

[17] C. W. J. Weston, "Support vector machines for multi-class pattern recognition," *Technical Report CSD-TR-98-04, Royal Holloway, University of London, 1998*, 1999.

[18] V. Stevanović, R. Trottier, C. Musgrave, F. Therrien, A. Holder, and P. Graf, "Predicting kinetics of polymorphic transformations from structure mapping and coordination analysis," *Phys. Rev. Materials*, vol. 2, p. 033802, Mar 2018. [Online]. Available: https://link.aps.org/doi/10.1103/PhysRevMaterials.2.033802

[19] "Pylada," https://github.com/pylada/pylada-polymorph-pathfinder.

[20] F. P. Bundy and J. S. Kasper, "Hexagonal diamond—a new form of carbon," *The Journal of Chemical Physics*, vol. 46, no. 9, pp. 3437–3446, 1967. [Online]. Available: https://aip.scitation.org/doi/abs/10.1063/1.1841236

[21] W. Utsumi and T. Yagi, "Formation of hexagonal diamond by room temperature compression of graphite," *Proceedings of the Japan Academy, Series B*, vol. 67, no. 9, pp. 159–164, 1991.

[22] C. L. Guillou, F. Brunet, T. Irifune, H. Ohfuji, and J.-N. Rouzaud, "Nanodiamond nucleation below 2273k at 15gpa from carbons with different structural organizations," *Carbon*, vol. 45, no. 3, pp. 636–648, 2007. [Online]. Available: http://www.sciencedirect.com/science/article/pii/S0008622306005057

[23] F. Isobe, H. Ohfuji, H. Sumiya, and T. Irifune, "Nanolayered diamond sintered compact obtained by direct conversion from highly oriented graphite under high pressure and high temperature," *Journal of Nanomaterials*, vol. 2013, p. 6, 2013. [Online]. Available: http://dx.doi.org/10.1155/2013/380165

[24] Z. Pan, H. Sun, Y. Zhang, and C. Chen, "Harder than diamond: Superior indentation strength of wurtzite bn and lonsdaleite," *Physical Review Letters*, vol. 102, no. 5, p. 055503, 2009. [Online]. Available: https://link.aps.org/doi/10.1103/PhysRevLett.102.055503

[25] B. Kulnitskiy, I. Perezhogin, G. Dubitsky, and V. Blank, "Polytypes and twins in the diamond-lonsdaleite system formed by high-pressure and high-temperature treatment of graphite," *Acta Crystallographica Section B*, vol. 69, no. 5, pp. 474–479, 2013. [Online]. Available: https://doi.org/10.1107/S2052519213021234

[26] A. Yoshiasa, Y. Murai, O. Ohtaka, and T. Katsura, "Detailed structures of hexagonal diamond (lonsdaleite) and wurtzite-type bn," *Japanese Journal of Applied Physics*, vol. 42, no. Part 1, No. 4A, pp. 1694–1704, 2003. [Online]. Available: http://dx.doi.org/10.1143/JJAP.42.1694

[27] D. J. Erskine and W. J. Nellis, "Shock-induced martensitic phase transformation of oriented graphite to diamond," *Nature*, vol. 349, no. 6307, pp. 317–319, 1991. [Online]. Available: https://doi.org/10.1038/349317a0

[28] C. G. Salzmann, B. J. Murray, and J. J. Shephard, "Extent of stacking disorder in diamond," *Diamond and Related Materials*, vol. 59, pp. 69–72, 2015. [Online]. Available: http://www.sciencedirect.com/science/article/pii/S0925963515300388

[29] F. P. Bundy, W. A. Bassett, M. S. Weathers, R. J. Hemley, H. U. Mao, and A. F. Goncharov, "The pressure-temperature phase and transformation diagram for carbon; updated through 1994," *Carbon*, vol. 34, no. 2, pp. 141–153, 1996. [Online]. Available: http://www.sciencedirect.com/science/article/pii/0008622396001704

[30] N. Dubrovinskaia, L. Dubrovinsky, F. Langenhorst, S. Jacobsen, and C. Liebske, "Nanocrystalline diamond synthesized from c60," *Diamond and Related Materials*, vol. 14, no. 1, pp. 16–22, 2005. [Online]. Available: http://www.sciencedirect.com/science/article/pii/S0925963504002225

[31] A. V. Kurdyumov, V. F. Britun, V. V. Yarosh, A. I. Danilenko, and V. B. Zelyavskii, "The influence of the shock compression conditions on the graphite transformations into lonsdaleite and diamond," *Journal of Superhard Materials*, vol. 34, no. 1, pp. 19–27, 2012. [Online]. Available: https://doi.org/10.3103/S1063457612010029

[32] P. Németh, L. A. J. Garvie, T. Aoki, N. Dubrovinskaia, L. Dubrovinsky, and P. R. Buseck, "Lonsdaleite is faulted and twinned cubic diamond and does not exist as a discrete material," *Nature Communications*, vol. 5, no. 1, p. 5447, 2014. [Online]. Available: https://doi.org/10.1038/ncomms6447

[33] A. Togo and I. Tanaka, "First principles phonon calculations in materials science," *Scripta Materialia*, vol. 108, pp. 1–5, 2015. [Online]. Available: http://www.sciencedirect.com/science/article/pii/S1359646215003127

[34] Y. Hinuma, G. Pizzi, Y. Kumagai, F. Oba, and I. Tanaka, "Band structure diagram paths based on crystallography," *Computational Materials Science*, vol. 128, pp. 140 – 184, 2017. [Online]. Available: http://www.sciencedirect.com/science/article/pii/S0927025616305110

[35] A. Togo and I. Tanaka, "`Spglib`: a software library for crystal symmetry search," *arXiv e-prints*, p. arXiv:1808.01590, Aug. 2018.

[36] L. Yang, K. C. Lau, Z. Zeng, D. Zhang, H. Tang, B. Yan, H. Gou, Y. Yang, Y. Xiao, D. Luo, S. Srinivasan, S. Sankaranarayanan, W. Yang, J. Wen, and H. kwang Mao, "Lonsdaleite: The diamond with optimized bond lengths and enhanced hardness," 2021. [Online]. Available: https://arxiv.org/abs/2111.09176

[37] F. P. Bundy, "Pressure-temperature phase diagram of elemental carbon," *Physica A: Statistical Mechanics and its Applications*, vol. 156, no. 1, pp. 169–178, 1989. [Online]. Available: http://www.sciencedirect.com/science/article/pii/0378437189901155

[38] R. Hoffmann, A. A. Kabanov, A. A. Golov, and D. M. Proserpio, "Homo citans and carbon allotropes: For an ethics of citation," *Angewandte Chemie International Edition*, vol. 55, no. 37, pp. 10 962–10 976, 2016. [Online]. Available: https://onlinelibrary.wiley.com/doi/abs/10.1002/anie.201600655

[39] V. A. Blatov, A. P. Shevchenko, and D. M. Proserpio, "Applied topological analysis of crystal structures with the program package topospro," *Crystal Growth & Design*, vol. 14, no. 7, pp. 3576–3586, 2014. [Online]. Available: https://doi.org/10.1021/cg500498k

[40] V. A. Blatov and D. M. Proserpio, "Topological relations between three-periodic nets. II. Binodal nets," *Acta Crystallographica Section A*, vol. 65, no. 3, pp. 202–212, May 2009. [Online]. Available: https://doi.org/10.1107/S0108767309006096

[41] V. A. Blatov, "Topological relations between three-dimensional periodic nets. I. Uninodal nets," *Acta Crystallographica Section A*, vol. 63, no. 4, pp. 329–343, Jul 2007. [Online]. Available: https://doi.org/10.1107/S0108767307022088
